# Framework Phylogeny, Evolution and Complex Diversification of Chinese Oaks

**DOI:** 10.3390/plants9081024

**Published:** 2020-08-13

**Authors:** Jia Yang, Yu-Fan Guo, Xiao-Dan Chen, Xiao Zhang, Miao-Miao Ju, Guo-Qing Bai, Zhan-Lin Liu, Gui-Fang Zhao

**Affiliations:** 1 College of Life Sciences, Northwest University, Xi’an 710069, China; guoyufan@stumail.nwu.edu.cn (Y.-F.G.); chenxiaodan@stumail.nwu.edu.cn (X.-D.C.); zhxiao@nwu.edu.cn (X.Z.); jumm089@nwu.edu.cn (M.-M.J.); bgq@ms.xab.ac.cn (G.-Q.B.); liuzl@nwu.edu.cn (Z.-L.L.); 2 Institute of Botany of Shaanxi Province, Xi’an 710061, China

**Keywords:** *Quercus*, phylogeny, evolution, rapid radiation, morphological divergence

## Abstract

Oaks (*Quercus* L.) are ideal models to assess patterns of plant diversity. We integrated the sequence data of five chloroplast and two nuclear loci from 50 Chinese oaks to explore the phylogenetic framework, evolution and diversification patterns of the Chinese oak’s lineage. The framework phylogeny strongly supports two subgenera *Quercus* and *Cerris* comprising four infrageneric sections *Quercus*, *Cerris*, *Ilex* and *Cyclobalanopsis* for the Chinese oaks. An evolutionary analysis suggests that the two subgenera probably split during the mid-Eocene, followed by intergroup divergence within the subgenus *Cerris* around the late Eocene. The initial diversification of sections in the subgenus *Cerris* was dated between the mid-Oligocene and the Oligocene–Miocene boundary, while a rapid species radiation in section *Quercus* started in the late Miocene. Diversification simulations indicate a potential evolutionary shift on section *Quercus*, while several phenotypic shifts likely occur among all sections. We found significant negative correlations between rates of the lineage diversification and phenotypic turnover, suggesting a complex interaction between the species evolution and morphological divergence in Chinese oaks. Our infrageneric phylogeny of Chinese oaks accords with the recently proposed classification of the genus *Quercus*. The results point to tectonic activity and climatic change during the Tertiary as possible drivers of evolution and diversification in the Chinese oak’s lineage.

## 1. Introduction

Species diversity is a key element of biodiversity on earth [1,2]. Reconstructing the evolutionary history of species-rich taxa provides fundamental insights about the forces shaping biodiversity [3,4,5]. Previous research targeting various taxa suggest that complex interactions, including abiotic elements (e.g., geohistorical and climatic fluctuations), biological factors (e.g., random genetic drift, introgression and life-history characteristics of organisms), and stochastic processes, could promote genetic turnover, evolution, and speciation [6,7,8,9,10,11]. Furthermore, these interactions have been hypothesized to correlate with evolutionary trade-offs in organisms’ investment strategies and, as a consequence, they can potentially shape the patterns of the morphological divergence and diversification rates across species evolution [12].

*Quercus* L. (Fagaceae) exhibits spectacular levels of species richness among angiosperms. Oaks are considered keystone species and they constitute the highest biomass to forest ecosystems in the Northern Hemisphere [13,14,15]. Central America and Mexico are a center of recent oak diversification, where the diverse oak species are demonstrated to be originated from North American oak clades through rapid adaptations and niche transitions [16]. Despite the utmost ecological importance of oak species, their classification has been a challenge that started in the 1800s and still continues today [17]. This challenge has resulted in various classification schemes based on morphology or on molecular data, and can be attributed to the complex evolutionary history of oak species, which is characterized by phenotypic similarity, reticulate evolution, and frequent hybridization [7,10,13,18,19,20,21,22]. Chinese oaks have not been the exception to this problem and have traditionally been classified into two genera or subgenera (*Quercus* and *Cyclobalanopsis*) based on phenotypic characters [23,24,25]. Recent work using genome data has made significant progress in the classification at the infrageneric level of genus *Quercus* [26]. In the updated classification, genus *Quercus* is separated into subgenera *Quercus* and *Cerris*, which correspond to the New and the Old World oaks, respectively. Subgenus *Quercus* encompasses five infrageneric sections: the white oaks (section *Quercus*), red oaks (section *Lobatae*), intermediate oaks (section *Protobalanus*), live oaks (section *Virentes*) and a relictual group with two endemic oak species (section *Ponticae*). Subgenus *Cerris* comprises three infrageneric groups, of which sections *Ilex* and *Cerris* are exclusively distributed across Eurasia, while section *Cyclobalanopsis* is entirely found in Asia [26,27]. However, for the Chinese oaks, although the DNA barcoding approach had been adopted to explore infrageneric groups and species boundaries among some Chinese oak species [28,29], to date a phylogenetic test assessing the Chinese oak’s lineage based on molecular data is lacking.

China hosts ca. 130 oak species according to *Flora of China* and is considered the diversity center of Eurasian oak species [23]. Fossil records and recently published molecular research suggest that the evergreen oak species of sections *Ilex* and *Cyclobalanopsis* probably originated in Southwestern China [30,31,32]. In terms of distribution, the Chinese oak species range from the eastern Himalaya to Taiwan Island, and from northeastern China to Hainan Island in subtropical and temperate areas, accounting for a prominent part of woody vegetation in East Asia and thus reflecting a remarkable ecological flexibility [33].

Despite the ecological and evolutionary importance of Chinese oaks, the diversification patterns and evolutionary history of the Chinese oak’s lineage are not well estimated. A series of phylogeographic research on some widespread Chinese oak species suggest that geographical distribution and climate change during the past geological periods could have affected the intra- and interspecific genetic variation of these species [34,35,36,37,38,39,40,41]. Additionally, research on Chinese oak species seems to imply that adaptation to different environments may influence genetic diversity, species turnover, and thus be relevant for the implementation of conservation strategies [32,42]. Given that a well-resolved phylogeny could provide a framework to understand diversification patterns and the evolutionary history of plant species [16], it is imperative to generate a framework phylogeny of the Chinese oaks to understand the evolutionary process and diversification pattern of this species-rich group.

The purposes of this study are: (1) to generate a phylogenetic framework and evolutionary history for 50 Chinese oak species (Table 1) based on five chloroplast regions and two nuclear loci, (2) to use simulations to understand the lineage diversification patterns and multiple traits-based evolution of the Chinese oak species under study, and (3) use simulation data to explore the potential association between the lineage diversification and phenotypic evolution in the Chinese oak’s lineage.

## 2. Results

### 2.1. Sequence Information of the Chinese Oaks

The DNA sequence alignment for the five plastid fragments ranged from 289 (*psb*A-*trn*H) to 734 bp (*mat*K) while the two nuclear genes were 332 and 355 bp for the internal transcribed spacer (ITS) and stress-associated protein gene (SAP), respectively (Table S1). All designed primers for the seven loci showed high amplification rates across the 50 Chinese oak species except for one species (*Quercus macrocalyx*), in which the *psb*A-*trn*H and ITS regions failed to produce results after repetitive PCR sequencing three times. The number of successfully sequenced individuals for the two nuclear loci was smaller than the samples obtained with the five chloroplast primer pairs due to the species-specific characteristics of SAP gene and the removal of potential ITS pseudogenes (Table S1).

The three genetic datasets (plastid dataset, nuclear dataset and combined plastid–nuclear data matrix) contained 239, 205 and 184 sequences, respectively, from 49 oak species for subsequent phylogenetic analyses. The maximum frequency of derived mutations (MFDM) tests among the Chinese oak species suggested no significant deviation from neutral evolution for the seven genetic loci (Table S1). An examination of chloroplast and nuclear data showed that the Chinese oaks have high levels of genetic diversity; similar results were obtained for the identified sections *Quercus, Ilex*, and *Cyclobalanopsis.* An exception to this pattern was the chloroplast dataset for section *Cerris*, which showed moderate genetic diversity; however, the nuclear data for this section also indicated high levels of genetic diversity (Table S2).

### 2.2. Infrageneric Phylogeny of the Chinese Oak’s Lineage

**Figure 1:**
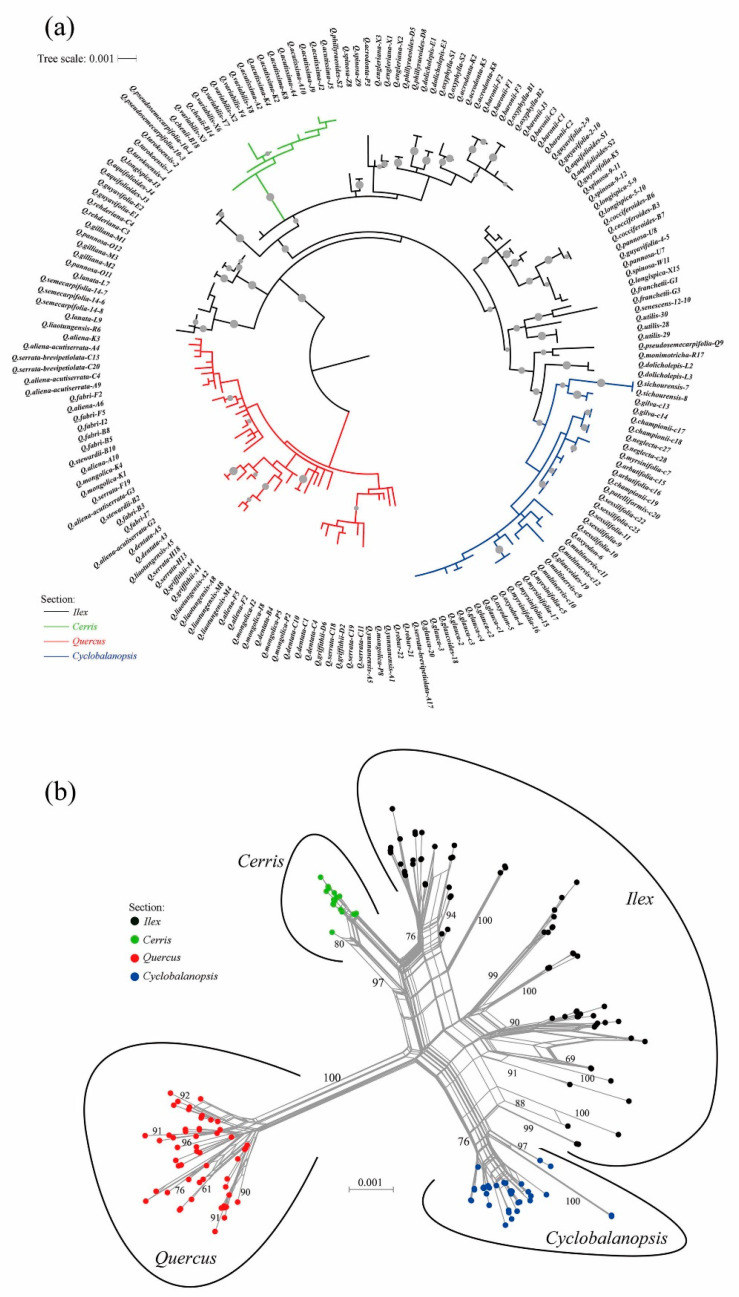
Infrageneric phylogeny of Chinese oak species inferred with (**a**) maximum likelihood and (**b**) neighbor-net methods using combined chloroplast–nuclear dataset. The four identified sections in the Chinese oak’s lineage are color-coded. Gray circles on branches of the maximum likelihood tree indicate bootstrap values > 60, and bootstrap confidence > 60 were showed for major clusters on the neighbor-net network.

Two major clades of the Chinese oak’s lineage were identified based on the maximum likelihood and neighbor-net analyses of the three genetic datasets. These two groups correspond to the deep divergence between the New World (subgenus *Quercus*) and the Old World oak clades (subgenus *Cerris*) (Figure 1, Figures S1 and S2). The New World oak clade comprised all Chinese white oaks (*Quercus* section *Quercus*) with strong bootstrap supports (>95). The rest comprised species from sections *Cerris*, *Ilex* and *Cyclobalanopsis*, clustered within the Old World oak clade. Section *Cerris* formed a monophyletic group with a strong bootstrap support (>90); however, it was unexpectedly nested within section *Ilex* (Figure 1a). Additionally, the later section (in chloroplast-based and chloroplast–nuclear-based phylogeny, Figure 1a and Figure S1) and section *Cyclobalanopsis* (in nuclear-based phylogeny, Figure S2) were not monophyletic. The best infrageneric framework uses a concatenated plastid–nuclear dataset, particularly the neighbor-net phylogenetic relationship that takes into account conflicting signals between chloroplast and nuclear loci, and clearly recovered the four proposed sections of the Chinese oak species according to the newly updated classification of the genus *Quercus* (Figure 1b).

Of the 50 Chinese oaks with two or more samples, 13 were found monophyletic on the best maximum likelihood phylogeny. Within sections *Ilex* and *Cyclobalanopsis*, the monophyletic oaks are endemic species with narrow distribution ranges (e.g., *Quercus cocciferoides*, *Q. utilis*, *Q. tarokoensis*, *Q. arbutifolia* and *Q. sichourensis*) or species with samples collected from a single location (e.g., *Q. semecarpifolia* and *Q. gilva*). Within section *Cerris*, the widespread *Q. acutissima* was monophyletic with a moderate support value (=57), suggesting possible genetic isolation with its sympatric sibling (*Q. variabilis*). By contrast, individuals of the Chinese white oaks from section *Quercus* were mixed together and no monophyly of a white oak species was supported by the phylogenetic relationships, indicating unclear species boundaries among these closely related species (Figure 1a; Table 1).

### 2.3. Evolutionary History of the Chinese Oaks

**Figure 2:**
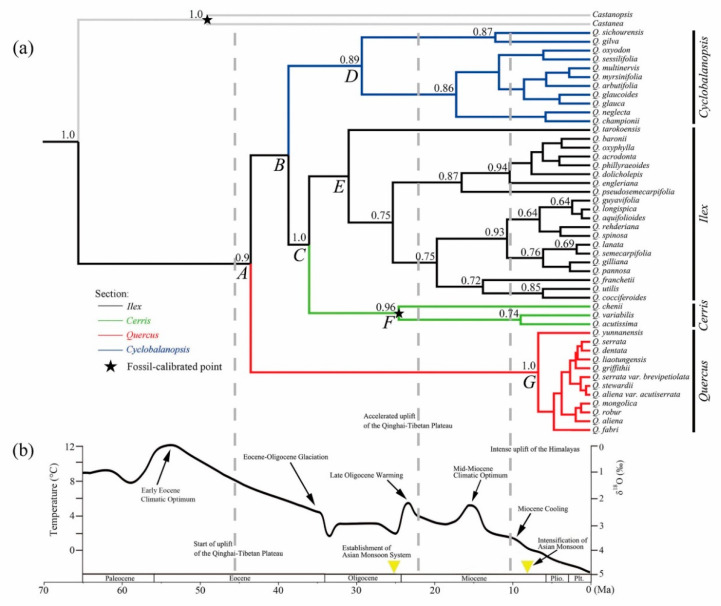
(**a**) Fossil-calibrated evolutionary history of the Chinese oaks and (**b**) geohistorical and climate events that possibly impacted on evolution of the Chinese oak’s lineage during the Cenozoic era [43,44]. Branches of the Bayesian evolutionary tree are colored according to the four identified sections. Black stars indicate fossil-calibrated points used in molecular dating. Posterior probabilities with values > 0.60 are shown near the nodes. Black curve in (**b**) indicates the global trends of temperature and average oxygen isotope from Paleocene to Pleistocene. Gray dashed lines and yellow triangles mirror the proposed dynamics of the Qinghai-Tibetan Plateau (QTP)-Himalaya and Asian monsoon systems, respectively.

Bayesian evolutionary analyses provided a concordant infrageneric phylogeny for the Chinese oak’s lineage at the species level (Figure 2). Based on two fossil-calibrated time points, the evolutionary tree indicated that the divergence between the *Quercus* ancestor and the outgroup occurred at about 65.54 Ma with a 95% highest posterior density (95% HPD) around 46.43–95.56 Ma. The initial divergence between the Old World oak clade and New World oak clade was estimated at 43.54 Ma (95% HPD: 25.02–64.92 Ma) (node A in Figure 2; Table 2). Within the Old World oak clade, the first split (node B) happened between the ring-cupped oak clade (section *Cyclobalanopsis*) and the *Ilex*-*Cerris* cluster at about 38.69 Ma with a moderate posterior probability (PP = 0.58); this event was closely followed by the divergence between sections *Ilex* and *Cerris* around 36.04 Ma (95% HPD: 28.37–52 Ma, node C). The crown ages of sections *Cyclobalanopsis* (node D) and *Ilex* (node E) were close to 29.29–30.97 Ma, while the *Cerris* crown age (node F) was estimated to be 24.56 Ma (95% HPD: 18.47–30.44 Ma). For the white oak clade (section *Quercus*), the lineage diversification of this group (node G) was inferred at about 6.66 Ma with a 95% HPD: 2.61–15.91 Ma (Figure 2; Table 2).

### 2.4. Lineage Diversification and Phenotypic Evolution of the Chinese Oak Species

**Figure 3:**
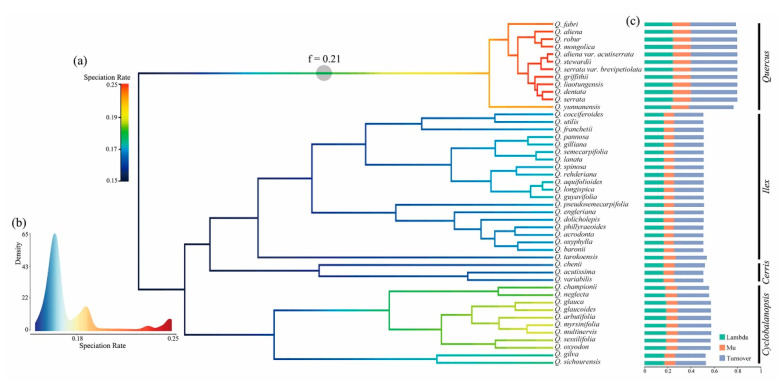
(**a**) Simulation on the lineage diversification of the Chinese oaks reveals varying diversification rates among the Chinese oak species and an evolutionary shift (Bayesian factor = 0.21) denoted by a gray circle on section *Quercus* clade. (**b**) Histogram distribution shows the variant density of speciation rates in Chinese oaks. (**c**) Bar charts of tip rates (lambda, mu and turnover rates) of lineage diversification for the Chinese oak species on the phylorate tree.

Diversification simulations showed varying speciation rates for the phylogeny of the 46 Chinese oak species studied. The phylorate tree suggested only one evolutionary shift (Bayesian factor = 0.21) with an increased speciation rate (mean: 0.2496 species/Myr) on the stem of the Chinese white oaks (section *Quercus*). Within the Old World oak clade, the ring-cupped oaks from section *Cyclobalanopsis* showed a slightly higher speciation rate (mean: 0.1899 species/Myr) compared with oaks from sections *Ilex* (mean: 0.1759 species/Myr) and *Cerris* (mean: 0.1730 species/Myr) (Figure 3; Table 2). Analyses of terminal diversification rates, including the mean speciation rate (lambda), extinction rate (mu) and turnover rate (lambda + mu), indicated that the diversification rate of the Chinese white oak clade was significantly higher than the Old World oak clade, while sections *Ilex* and *Cerris* had similar diversification rates (Figure 3; Table 2). Pairwise comparisons of mean speciation rates (lambda rate) among the four identified sections showed significant differences between the New World oak clade (section *Quercus*) and sections *Cerris*, *Ilex* and *Cyclobalanopsis*, which was confirmed with a Bonferroni *t*-test with 5000 random samples. Similarly, within the Old World oak clade, significant speciation rate differences were found between sections *Cerris* and *Cyclobalanopsis* and sections *Ilex* and *Cyclobalanopsis*; however, no differences in the simulated speciation rates were found between sections *Cerris* and *Ilex* (Table S3).

**Figure 4:**
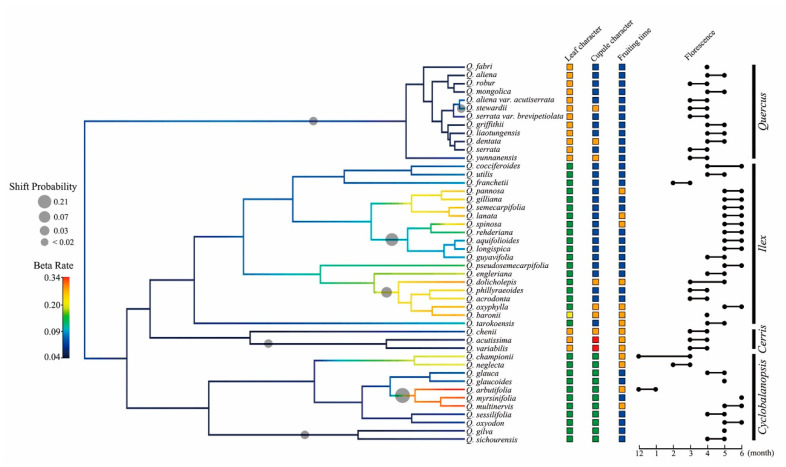
Simulation of the phenotypic evolution of the Chinese oaks suggests heterogeneous rates in trait-based evolution and several phenotypic transitions in the Chinese oak’s lineage. Gray circles on branches denote potential phenotypic shifts with varying Bayesian probabilities. Trait characteristics of the tip species are coded with colored squares as follows: leaf character-deciduous (orange), evergreen (green) and semievergreen (yellow); cupule character-ovate bract (blue), lanceolate bract (orange), subulate bract (red) and ring shaped bract (green); fruiting time-present year (blue) and the following year (orange). Dumbbell charts demonstrate the florescent periods of the tip species on the phylogeny.

Simulations of global phenotypic diversification on PC1 traits indicated seven trait-based shifts with probabilities ranging from <0.02 to 0.21 across the Chinese oak’s lineage (Figure 4). Major transitions of phenotypic traits with high probability were found within the Old World oak clade, and generally corresponded to species clusters with increased phenotypic diversification rates (beta rates). In contrast to the lineage diversification results (Figure 3), the Chinese white oaks (section *Quercus*) showed relatively low beta rates; also, two shifts with low Bayesian probabilities (<0.02) were identified on the stem of this section and on the endemic *Q. stewardii* (Figure 4). A Bonferroni *t*-test for pairwise comparison of mean rates in phenotypic evolution (beta rate) among the four identified Chinese oak sections indicated no significant differences between the two deciduous sections *Quercus* and *Cerris*. In contrast, the evergreen sections *Ilex* and *Cyclobalanopsis* showed significant differences against the deciduous sections *Quercus* and *Cerris* in phenotypic evolution. A significant difference in beta rates was also found between sections *Ilex* and *Cyclobalanopsis* (Table S3).

**Figure 5:**
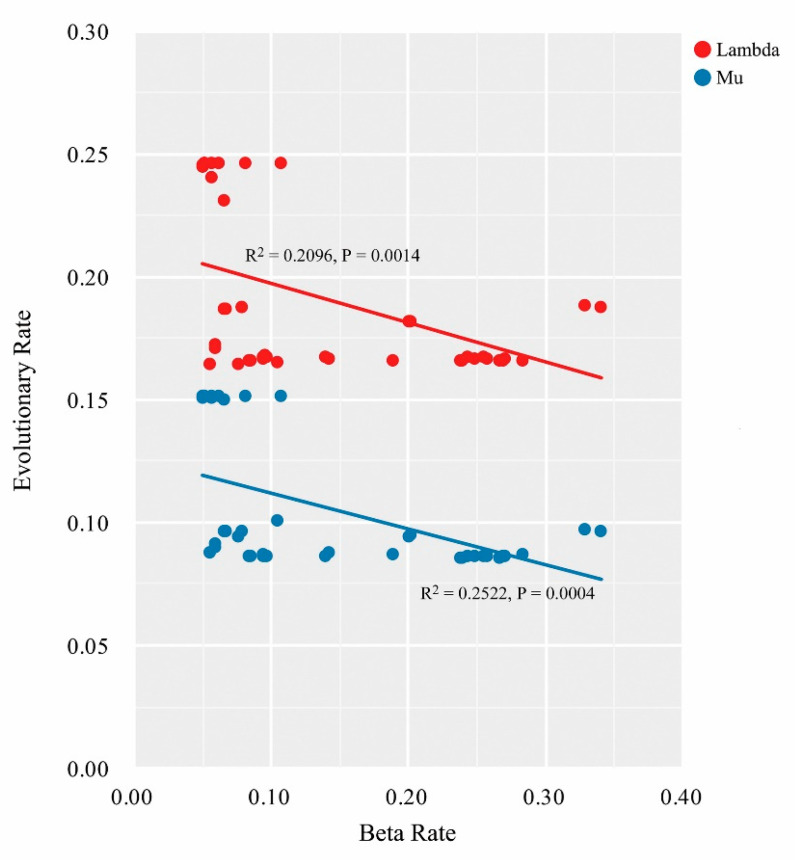
Relationships between the tip rates in the phenotypic evolution (beta rates) and lineage diversification (lambda and mu rates) of the Chinese oak’s lineage under a generalized linear regression.

After using generalized linear regressions, the association analyses suggested significant and negative correlations between the tip rates in the phenotypic divergence and lineage diversification (lambda vs. beta: R^2^ = 0.2096, P = 0.0014; mu vs. beta: R^2^ = 0.2522, P = 0.0004) for the 46 Chinese oak species in diversification simulations (Figure 5).

## 3. Discussion

The Chinese oak’s lineage is characterized by a high species richness and diverse species morphology, which are proposed as potential origins of oak species diversity [23,30]. Despite this, there is no current molecular-based phylogenetic framework to understand the evolution of the Chinese oaks; one of the main reasons for this outcome is, in part, that previous research has focused on oak species from areas outside China. In this study, we integrated chloroplast and nuclear data from 50 Chinese oak species to investigate their phylogenetic relationships, evolutionary history and diversification patterns. Our results provide an infrageneric phylogeny for the Chinese oaks and point to the potential impact of past geological and climatic changes on the evolution and diversification of this species-rich lineage.

### 3.1. Infrageneric Phylogeny of Chinese Oaks

The phylogenetic reconstruction obtained in this study identified two major clades (subgenera *Cerris* and *Quercus*) with four sections—*Quercus*, *Ilex*, *Cerris* and *Cyclobalanopsis*—for the Chinese oak’s lineage (Figure 1 and Figure 2). Although a previous study based on chloroplast loci did not provide enough support for these sections [29], the use of a combined plastid–nuclear dataset improved the infrageneric resolution of the Chinese oak phylogeny. An examination of the phylogenetic trees based on separate plastid and nuclear datasets revealed a phylogenetic incongruence, which has previously been documented for oaks [45,46]. Specifically, the concatenated chloroplast dataset showed sections *Cerris* and *Cyclobalanopsis* as monophyletic but nested within section *Ilex*, while the phylogenetic tree based on nuclear data revealed a very different tree structure with a monophyletic section *Cerris* and a polyphyletic section *Cyclobalanopsis*, both nested within section *Ilex* (Figures S1 and S2). Apart from the limited genetic data used in this study, this incongruence is proposed to be affected by taxonomic decoupling and geographic restraint in the plastid genealogy of oak species; thus, the plastid sequence data is suggested to be less useful for estimating species phylogenetic relationships in oaks [27,46]. However, the conflict between the two datasets seems to be recovered by the neighbor-net method in this study, as the neighbor-net network based on combined plastid–nuclear data strongly shows the presence of two subgenera and four infrageneric species groups for the Chinese oak’s lineage (Figure 1b). Overall, our results are consistent with the *Quercus* infrageneric phylogeny proposed by Denk et al. (2017), which is based on a large number of DNA sequences from a comprehensive sampling of European and American oaks [20,21,27,47,48]; furthermore, this updated infrageneric phylogeny is fully consistent with an oak genomic landscape analysis [26].

The infrageneric phylogeny of the Chinese oaks that we obtained shows that only 13 from the 50 collected species were monophyletic, reflecting an extensive introgression and consequent difficulty in defining species boundaries, especially among closely related species. Most of the monophyletic species are endemic in sections *Ilex* and *Cyclobalanopsis*; in contrast, not a single monophyletic species was resolved in section *Quercus* (subgenus *Quercus*) (Figure 1). These results are likely the consequence of the complex gene flow among closely related species which suggests an incomplete lineage isolation [28,34]. Similar interspecific gene flow patterns have been observed among some close relatives of sections *Ilex* and *Cyclobalanopsis* [49,50]. Moreover, it is likely that the limited genetic data used in this study is not enough to identify the biological boundaries and phylogenetic relationships among closely related species; thus, additional data will be necessary to identify monophyletic groups. Ideally, the species delimitation problem in oaks could be solved using genome information; however, recent studies based on plastome and genomic data have revealed similar difficulties to those faced in this study. Indeed, several authors have highlighted that, even with the use of genome data, finding monophyletic groups for some oak species is challenging [26,46].

### 3.2. Evolutionary History of the Chinese Oaks

Several research groups have studied the evolutionary history of *Quercus* and have estimated dissimilar divergence times for different oak groups, e.g., [16,48,51,52]. Based on a robust phylogeny of the genus *Quercus,* Hipp et al. (2020) obtained older crown times, for all infrageneric clades, than previously reported [26]. In evolutionary analyses, different factors could cause discrepancies in the estimation of molecular dating such as: the calibration time points (e.g., fossil-calibrated point on a crown age or a stem age), the phylogenetic signals among different genetic datasets (e.g., multiple genetic datasets considered in this study), and the inclusion of species from different lineages [16,45,48,51]. In fact, in our study, the limited genetic data and calibrated points could have introduced some bias to the molecular dating. For example, the minimum and maximum age range for section divergence and lineage diversification were broad for some clades (Figure 2; Table 2); these broad age range intervals could be the result of conflicts between the phylogenetic signals from the plastid and nuclear datasets. Despite this situation, the combined chloroplast and nuclear dataset using a coalescent method produced a phylogenetic framework that is consistent with the most recent phylogeny for the genus *Quercus* (Figure 1).

Our results indicate that the initial divergence of the Chinese oak’s lineage, that is the split between the two subgenera *Quercus* and *Cerris*, possibly happened around the mid-Eocene, 43.54 Ma (node A in Figure 2; Table 2) when the Qinghai-Tibetan Plateau (QTP) began to uplift [43,53]. Contrasting opinions also suggest that the elevation of the QTP reached about 4000–5000 m at the mid-Eocene epoch (*ca.* 45 Ma) [54]. This might have caused a decrease in temperature during mid- to late Eocene, leading to the Eocene–Oligocene glaciation [43,44]. Climatic and geological fluctuations during this period likely caused ecological differentiation between low and high latitude regions, triggering the divergence between ancestors of the Old World oak clade (subgenus *Cerris*) (from a mid- to low latitude), and the New World oak clade (subgenus *Quercus*), distributed at a high latitude [27,45]. Within the Old World oak clade, the sections recognized in our work could have arisen before the late Eocene, thus it is possible that the low temperature with an arid environment during the Eocene–Oligocene glaciation could have promoted the split of the humidity-adapted *Cyclobalanopsis* lineage (node B) and the arid-tolerant oaks from section *Ilex* (node C). Evidence for this inference is the appearance of fossil records of sections *Ilex* and *Cyclobalanopsis* in Asia from the mid- to late Eocene [23,55]. According to our reconstruction, the initial diversification within the two evergreen clades (sections *Cyclobalanopsis* and *Ilex*, nodes D and E in Figure 2; Table 2) probably took place during the early to mid-Oligocene; during this glacial period, the relatively stable climates likely promoted long distance migration (such as the European holly oaks, which originated from the East Asian oak lineage and migrated via the Tibet-Himalaya corridor) [32] and the geographical isolation of some evergreen oaks (e.g., *Q. sichourensis* and *Q. tarokoensis* in this study). We estimated that most species from sections *Ilex* and *Cyclobalanopsis* differentiated around the early to mid-Miocene when the intense uplift of the QTP and Himalaya took place; during this time, temperature fluctuations from the climatic optimum to cooler temperatures could have promoted the increase in genetic variation and ecological differentiation of species in these two sections [31,43]. Furthermore, fossil evidence from the Miocene consisting of abundant and morphologically diverse evergreen oak species supports the diversification of sections *Cyclobalanopsis* and *Ilex* during this time period in Asia [23]. Within section *Cerris*, the presence of fossil records in Western Eurasia appears to support our estimation that the first divergence of this group probably happened around the Oligocene–Miocene boundary (node F in Figure 2; Table 2). However, a revised fossil record of section *Cerris* in the Russian Far East suggests that the differentiation of this section could have occurred in the early Oligocene [56]. If the divergence of *Cerris* oaks took place during the late Oligocene, then it is possible that the establishment of the Asian monsoon system, with increasing temperatures, might have triggered the differentiation of deciduous oaks in this section. Independently of the origin, the *Cerris* oaks are currently the most diverse group in Western Eurasia with eight accepted species. In contrast to the early divergence between the New and Old World oak clades, species differentiation within section *Quercus* likely took place in the late Miocene (node G in Figure 2; Table 2). Species diversification in this section could be the result of descending temperatures and an intensification of the Asian monsoon system during the late Miocene [43]. Within section *Quercus,* it has been suggested that the Eurasian Roburoid oaks became established around the mid-Miocene period from ancestors of the North American *Prinoideae* and *Albae* clades, and then split into East Asian and Western Eurasian lineages since ca. 10 Ma [26], corresponding closely to our time estimation. Similar to previous research on American and Eurasian oak species [16,48], our analyses also support a rapid radiation of the Chinese white oak clade (section *Quercus*) from the late Tertiary to early Quaternary (Figure 2).

### 3.3. Complex Diversification of the Chinese Oak’s Lineage

In general, diversification simulations suggest homogeneous lineage diversification rates (mean lambda: 0.1731 species/Myr) for the 46 oak species studied, with the exception of a possible evolutionary shift (non-core shift) for the Chinese white oak clade (section *Quercus*) (mean lambda: 0.2496 species/Myr) (Figure 3; Table 2). In a study of global oak evolution based on genomic data, Hipp et al. (2020) found a similar pattern for species in section *Quercus*; their results showed an evolutionary shift with an increased speciation rate for a group of white oak species referred to as the Roburoid clade (which included some Chinese white oak species) [26]. This evolutionary shift in section *Quercus* could be the result of different evolutionary dynamics between the New and Old World oak clades, as fossil evidence suggests that the ancestors of these two clades originated in the Nearctic and Palearctic Indomalayan regions, respectively [27,45]; thus, it is possible that species in these clades adapted to the different geological and ecological conditions associated with their distinct regions of origin. Furthermore, different species diversification patterns and migration routes could have led to disparate evolutionary conditions for species in sections *Quercus* (the New World oak clade) and other sections (the Old World oak clade) [16,26]. Another plausible explanation for the diversification differences between subgenera *Quercus* and *Cerris* is that the rapid radiation of the white oak clade since the late Miocene (Figure 2) might generate a conserved evolutionary configuration during relatively short speciation processes [57]. Within subgenus *Cerris*, the mean diversification rate for the ring-cupped oak clade (section *Cyclobalanopsis*) is slightly higher than sections *Ilex* and *Cerris* (Table 2), but it is not identified as a shift by the Bayesian models. Given the small fraction of species from section *Cyclobalanopsis* included in the Bayesian analysis of macroevolutionary mixtures (BAMM) (Table S4), this result requires further investigation with a more comprehensive sampling.

Simulations on global phenotypic evolution (PC1) suggest a low morphological diversification rate in section *Quercus*, mirroring the similarity of the morphologies among Chinese white oaks; however, this outcome could be a reflection of the limited phenotypic difference information provided by the selected morphological characters (Figure 4; Table 1 and Table S5). Alternatively, the low morphological diversification rate in section *Quercus* could be the result of their recent origin (Figure 2) and associated absence of strong reproductive isolation barriers, which could facilitate extensive hybridization. An example that supports the latter statement comes from studies in Roburoid oaks (a subgroup that belongs to section *Quercus*)*,* such as *Q. robur* and *Q. petraea*, which appear to have frequent interspecific introgressions [58]. The taxonomic confusion in the Roburoid clade is proposed to be the result of morphological convergence and niche conservatism [26]. By contrast, Chinese oak sections belonging to the Old World oak clade show diverse rates in phenotypic diversification; possible shifts of morphological traits found on several clusters in sections *Ilex* and *Cyclobalanopsis* correspond to the differences in traits of phenotypic characters, florescence and fruiting time (Figure 4; Table 1 and Table S5). Given the early divergence of the Old World oak clade (Figure 2; Table 2), potential reasons for a phenotypic turnover within this clade are its long evolutionary history, adaptation to heterogeneous environments, and divergence caused by migration and geographic disruptions [31,32,45].

Interestingly, the significant and negative linear relationships between the tip rates in the lineage diversification and phenotypic evolution of the Chinese oak’s lineage indicate that morphological traits tend to be more differentiated in oak species that have low diversification rates (Figure 5). Complex associations between morphological divergence and species diversification have been predicted in animals [59]. Although this process has rarely been investigated in plant groups, an early study on the species-rich clades Adoxaceae and Valerianaceae has revealed negligible correlations between the rates of diversification and morphological innovation [60]. For the Chinese oaks in this study, it is plausible that the increased rates of lineage diversification in section *Quercus* (Figure 3; Table 2) could be associated with a relatively high genetic variation (Table S2) in the genome (such as structural variation) [61], while this type of generic variation is not correlated with distinct morphology. Additionally, frequent introgressions among oak siblings likely induces the exchange of adaptive genes as well as reduces the phenotypic variability in section *Quercus* [58,62]. On the other hand, a study on the diversification patterns of the American oaks in a community assembly showed that the ecological divergence among closely related species from the same clade, accompanied by the niche convergence of species between distant clades, could trigger complex diversification patterns in oak lineages [57]. A third possibility for the negative correlations we found is that the intrinsic trade-offs between the functional traits and phenotypic traits in species investment strategies could generate multivariate associations with lineage diversifications [12,63]. According to this hypothesis, our negative correlations might be a predictor of the diversification process of the Chinese oak’s lineage. Yet, given the neutrality of the genetic data in this study (Table S1), future work integrating adaptation genes, functional traits, as well as ecological data may help us understand the complex associations of diversification patterns in the genus *Quercus* [64].

## 4. Material and Methods

### 4.1. Species Information and DNA Extraction

We collected 268 individuals representing 50 Chinese oak species covering the proposed infrageneric groups of Eurasian oaks. Species identifications were made based on their morphological descriptions in *Flora of China* (Table 1). Oak samples were collected from natural populations and arranged according to the identified taxonomy of the genus *Quercus* [26,27]. Of the 50 oaks studied, *Quercus robur* is a European oak species and was sampled from cultivation in Tacheng City, Xinjiang Province. Voucher specimens of the collected oak species were archived in the herbarium of the College of Life Sciences at Northwest University.

Fresh leaves were collected from adult oak trees and were dried with silica gel prior to DNA extraction. The total genomic DNA was isolated using the plant genomic DNA Kit from TIANGEN (Tiangen, Beijing, China) and stored at 4 °C for PCR amplification.

### 4.2. PCR Amplification and Molecular Data Processing

Five chloroplast DNA fragments (*psb*A-*trn*H, *mat*K, *ycf*1, *ycf*3-*trn*S and *mat*K-*trn*K), showing relatively high genetic variations in some Chinese oaks, were selected from the preliminary research [28]. Two nuclear regions, the internal transcribed spacer (ITS, partial regions) and a confirmed single-copy gene SAP (a predicted stress-associated protein gene) [28] were also sequenced for all samples collected. The primer information for these seven loci is listed in Table S1. PCR amplifications were performed in a PTC-2000 thermal cycler (MJ Research) following Yang et al’s method [65], and the PCR products were purified and sequenced by Sangon (Sangon Biotech, Shanghai, China). All obtained sequences for this study were deposited in GenBank with accession numbers: KX836866-KX838287, MT129797-MT130099, and MT131185-MT131230.

All DNA sequences obtained were checked and aligned in BioEdit 7.0.9.0 [66]. Ambiguous sites with poly-(A/T) structures detected in three plastid fragments (*psb*A-*trn*H, *ycf*3-*trn*S and *mat*K-*trn*K) and poly-C in the ITS region were deleted from the alignments. Insertion-deletions (indels) were treated as missing data and removed prior to the analyses. Following the criteria used by Yang et al. (2017), the obtained sequences of the ITS region were carefully examined to filter out potential pseudogenes, while functional ITS orthologues were retained for phylogenetic reconstruction [28]. Sequencing peaks of nucleotide sites with an overlap greater than 80% in the two nuclear loci were treated as heterozygous, and the ambiguity code was used to replace the overlapped sites according to the IUPAC (International Union of Pure and Applied Chemistry). For each alignment of plastid and nuclear data, a neutrality test using the maximum frequency of derived mutations (MFDM) method, with a 5% significance level, was performed to detect selection among the Chinese oak samples [67].

### 4.3. Reconstruction of Infrageneric Phylogeny

The individual alignments of the seven genetic regions provided poor information for deciphering the Chinese oaks infrageneric phylogeny (Figures S3 and S4); thus, the aligned genetic data were processed as three data matrices: (1) the concatenated chloroplast data matrix, (2) concatenated nuclear dataset, and (3) combined chloroplast–nuclear genetic data for phylogenetic tests. For the chloroplast (matrix 1) and nuclear (matrix 2) datasets, the global indices of genetic diversity (such as polymorphic and parsimony sites, gene and nucleotide diversity, and variance of segregating sites) for the Chinese oak’s lineage and each identified section were estimated using DnaSP 5.0 [68].

The ultrafast bootstrap approximation (UFBoot) implemented in the IQ-TREE software package was used to estimate the maximum likelihood (ML) phylogeny for the Chinese oak species under study [69,70]. Unrooted ML trees were constructed for the three genetic data matrices and for the individual gene alignments. The evolutionary models were initially evaluated by Bayesian schemes with the ModelFinder option and Akaike information criterion (AIC) implemented in the IQ-TREE for the three datasets. Phylogenetic trees were then estimated using the best-fit partition models with 1000 UFBoot replicates. Final trees were visualized in iTOL v4 [71].

The neighbor-net method was also used to estimate the genetic relationships among all oak samples in SplitsTree4 [72]. This method is an extension of the neighbor-joining method and quantifies the potential conflicting signals among multiple genetic loci for phylogenetic analyses. For heterozygous sites, all possible resolutions were averaged using the uncorrected P-distance, and 1000 bootstrap simulations were estimated for the neighbor-net network.

### 4.4. Molecular Dating

To estimate the evolutionary history of the Chinese oak species, we utilized a multispecies coalescent-based Bayesian analysis, as implemented in BEAST 1.8.4 [73], for the combined chloroplast–nuclear data matrix (matrix 3). Species samples from *Castanopsis* and *Castanea* were included as outgroups. The combined chloroplast–nuclear data matrix was partitioned into six input groups (*psb*A-*trn*H, *mat*K, *ycf*1, *ycf*3-*trn*S + *mat*K-*trn*K, ITS, SAP) according to the IQ-TREE partition results (Table S1), and their corresponding evolutionary models were used to estimate the Bayesian evolutionary tree. For the data matrix, 46 of the 50 Chinese oak species with more than two individuals were retained, and a “trait” file was compiled to assign the samples of each oak species. The Bayesian evolutionary tree was calculated using a relaxed clock model in a lognormal distribution and a linear coalescent species model with a birth–death speciation process. Heterozygous sites in the two nuclear regions were treated as polymorphisms with a modified setting (“useAmbiguities” = “true”) in the input file for estimation. Three independent runs were carried out with 8 × 10^8^ Markov chain Monte Carlo (MCMC) generations and sampled every 10^4^ generations to ensure an effective sample size (ESS > 200) and model convergence. We considered two fossil time points to calibrate the divergence of major groups among the Chinese oaks: (1) a normal divergence between genera *Castanopsis* and *Castanea* at a mean age of 45–55 Ma [74], and (2) a minimum split time at 23 Ma for the crown age of section *Cerris* (23–33 Ma) [48]. The final results of the three independent simulations were combined using LogCombiner, and a maximum clade credibility (MCC) tree with a posterior probability >0.8 was calculated after removing the first 20% simulations as burn-in. The fossil-calibrated MCC tree of the Chinese oak’s lineage was visualized in FigTree 1.4.2 from the BEAST software package. Major climatic and geological events during the Cenozoic era were derived to evaluate the possible abiotic triggers of divergence and diversification for the Chinese oak’s lineage [43,44].

### 4.5. Diversification Simulation

Based on the Bayesian evolutionary tree for the Chinese oak species, we simulated the patterns of lineage diversification by estimating the transitions of diversification rates using the time-varying speciation–extinction model in the Bayesian analysis of macroevolutionary mixtures (BAMM) program [75]. To account for our incomplete species sampling of Chinese oaks, a species-specific file indicating sampling fractions of the backbone (Table S4) was compiled to correct for a possible analytical bias during the simulations of lineage diversification. The R package “BAMMtools” was used to configure the prior parameters and downstream analyses of BAMM simulations [76]. The analyses were performed with 10^7^ MCMC generations and sampled every 1000 swap periods across four Markov chains for convergence.

The phenotypic diversification of the Chinese oak species was also evaluated using the trait-based model in the BAMM program. We assembled four species-specific traits including the leaf character, cupule character, mid-time of florescence and fruiting for the collected Chinese oak species (Table 1) based on the morphological descriptions in *Flora of China*. The four phenotypic traits represented in a numerical format were processed using a principal component analysis (PCA) (Figure S5), and the standard scores for each oak species on the first principal component (PC1, accounted for 51.6% of the variance in the PCA) (Table S5) were extracted and used to simulate the global traits-based diversification of the Chinese oak species using BAMM. Simulations were performed with 8 × 10^8^ MCMC generations and sampled every 4000 generations under four Markov chains.

For the identified infrageneric sections on the Chinese oaks phylogeny, a pairwise comparison of diversification rates from BAMM simulations was estimated using a Bonferroni *t*-test with 5000 random samples of each infrageneric section. The potential associations between the tip rates in the lineage diversification and phenotypic evolution of the Chinese oaks were assessed with a generalized linear regression. These statistical analyses were performed in R 3.6 [77].

## 5. Conclusions

In this study, we generated a framework phylogeny for 50 Chinese oak species based on chloroplast and nuclear data. Our infrageneric framework clearly identifies two subgenera (*Quercus* and *Cerris*) and four infrageneric sections (*Quercus*, *Cerris*, *Ilex* and *Cyclobalanopsis*), in agreement with the most recent phylogeny of the genus *Quercus* [27]. Additionally, the results support that the widely used traditional classification of Chinese oak species based on morphology needs to be revised. In terms of evolutionary history, our study suggests that the primary divergence of the Chinese oaks probably occurred at mid-Eocene, and was followed by the establishment of sections *Cerris*, *Ilex*, and *Cyclobalanopsis* in subgenus *Cerris* around the late Eocene to early Oligocene. Furthermore, we estimated that the rapid diversification of the Chinese white oaks from section *Quercus* probably began since the late Miocene. Using a temporal simulation model on diversification patterns, we found a potential evolutionary shift on the Chinese white oak clade, suggesting an evolutionary decoupling between the New World and Old World oaks at their initial divergence. Additionally, we identified several phenotypic shifts for all sections in the Chinese oak’s lineage, which suggests a complex history of diversification during the evolution of oak species. Given the ecological importance of the genus *Quercus*, this research provides evidence that integration of abiotic processes, including tectonic activity and climatic fluctuations during the Tertiary, have shaped the evolution and diversification of the Chinese oak’s lineage.

## Figures and Tables

**Table 1:** Taxon information and phenotypic features of the Chinese oak species used in this study.

Section	Taxon	Samples	Leaf Character	Cupule Character	Florescence Range	Fruiting Period with Florescence
*Quercus*	*Quercus aliena* Blume	9	Deciduous	Ovate bract	Apr.–May	Present year
	*Q. aliena* var. *acutiserrata* Maximowicz ex Wenzig	9	Deciduous	Ovate bract	Mar.–Apr.	Present year
	*Q. dentata* Thunb.	9	Deciduous	Lanceolate bract	Apr.–May	Present year
	*Q. fabri* Hance	9	Deciduous	Ovate bract	Apr.	Present year
	*Q. griffithii* Hooker and Thomson ex Miquel	6	Deciduous	Ovate bract	Apr.–May	Present year
	*Q. liaotungensis* Koidz. (or *Q. wutaishanica* Blume)	9	Deciduous	Ovate bract	Apr.–May	Present year
	*Q. mongolica* Fischer ex Ledebour	9	Deciduous	Ovate bract	Apr.–May	Present year
	Q. robur L. ^†^	2	Deciduous	Ovate bract	Mar.–Apr.	Present year
	*Q. serrata* Murray	8	Deciduous	Ovate bract	Mar.–Apr.	Present year
	*Q. serrata* var. *brevipetiolata* (A. DC.) Nakai	6	Deciduous	Ovate bract	Mar.–Apr.	Present year
	*Q. stewardii* Rehd.	3	Deciduous	Lanceolate bract	Mar.–Apr.	Present year
	*Q. yunnanensis* Franchet	3	Deciduous	Lanceolate bract	Mar.–Apr.	Present year
*Ilex*	*Q. acrodonta* Seemen	6	Evergreen	Ovate bract	Mar.–Apr.	Present year
	*Q. aquifolioides* Rehd. and Wils.	8	Evergreen	Ovate bract	May–Jun.	Present year
	*Q. baronii* Skan	9	Semievergreen	Lanceolate bract	Apr.	Following year
	***Q. cocciferoides* Hand.-Mazz.**	3	Evergreen	Ovate bract	Apr.–Jun.	Present year
	*Q. dolicholepis* A. Camus	6	Evergreen	Lanceolate bract	Mar.–May	Following year
	***Q. engleriana* Seemen**	3	Evergreen	Ovate bract	Apr.–May	Present year
	***Q. franchetii* Skan**	3	Evergreen	Ovate bract	Feb.–Mar.	Present year
	*Q. gilliana* Rehd. and Wils.	3	Evergreen	Ovate bract	May–Jun.	Present year
	*Q. guyavifolia* H. Leveille	8	Evergreen	Ovate bract	Apr.–May	Present year
	*Q. lanata* Smith	3	Evergreen	Ovate bract	May–Jun.	Following year
	*Q. longispica* (Hand.-Mazz.) A. Camus	6	Evergreen	Ovate bract	May–Jun.	Present year
	*Q. monimotricha* Hand.-Mazz.	4	Evergreen	Ovate bract	May–Jun.	Following year
	*Q. oxyphylla* (E. H. Wilson) Hand.-Mazz.	6	Evergreen	Lanceolate bract	May–Jun.	Following year
	*Q. pannosa* Hand.-Mazz.	4	Evergreen	Ovate bract	May–Jun.	Following year
	*Q. phillyraeoides* A. Gray	6	Evergreen	Ovate bract	Mar.–Apr.	Present year
	*Q. pseudosemecarpifolia* A. Camus	6	Evergreen	Ovate bract	May–Jun.	Present year
	***Q. rehderiana* Hand.-Mazz.**	3	Evergreen	Ovate bract	May–Jun.	Present year
	***Q. semecarpifolia* Smith**	3	Evergreen	Ovate bract	May–Jun.	Present year
	*Q. senescens* Hand.-Mazz.	10	Evergreen	Ovate bract	Mar.–May	Present year
	*Q. spinosa* David ex Franchet	15	Evergreen	Ovate bract	May–Jun.	Following year
	***Q. tarokoensis* Hayata**	4	Evergreen	Ovate bract	Apr.–May	Following year
	***Q. utilis* Hu and W. C. Cheng**	3	Evergreen	Ovate bract	Apr.–May	Present year
*Cerris*	***Q. acutissima* Carruth.**	9	Deciduous	Subulate bract	Mar.–Apr.	Following year
	*Q. chenii* Nakai	3	Deciduous	Lanceolate bract	Mar.–Apr.	Following year
	*Q. variabilis* Blume	6	Deciduous	Subulate bract	Mar.–Apr.	Following year
*Cyclobalanopsis*	***Q. arbutifolia* Hickel and A. Camus**	2	Evergreen	Ring shaped bract	Dec.–Jan.	Following year
	*Q. championii* (Bentham) Oersted	3	Evergreen	Ring shaped bract	Dec.–Mar.	Following year
	***Q. gilva* (Blume) Oersted**	5	Evergreen	Ring shaped bract	May	Present year
	*Q. glauca* (Thunberg) Oersted	7	Evergreen	Ring shaped bract	Apr.–May	Present year
	*Q. glaucoides* Schottky	2	Evergreen	Ring shaped bract	May	Present year
	*Q. macrocalyx* Hickel and A. Camus	2	Evergreen	Ring shaped bract	Mar.–Apr.	Present year
	*Q. multinervis* W. C. Cheng and T. Hong	4	Evergreen	Ring shaped bract	May–Jun.	Following year
	*Q. myrsinifolia* (Blume) Oersted	7	Evergreen	Ring shaped bract	Jun.	Present year
	***Q. neglecta* Schottky**	2	Evergreen	Ring shaped bract	Feb.–Mar.	Following year
	*Q. oxyodon* (Miquel) Oersted	3	Evergreen	Ring shaped bract	May–Jun.	Present year
	*Q. patelliformis* (Chun) Y. C. Hsu and H. W. Jen	2	Evergreen	Ring shaped bract	May–Jun.	Present year
	***Q. sessilifolia* (Blume) Schottky**	5	Evergreen	Ring shaped bract	Apr.–May	Present year
	***Q. sichourensis* Hu**	2	Evergreen	Ring shaped bract	Apr.–May	Present year

Species names in bold indicate monophyletic found on the maximum likelihood phylogeny in Figure 1; phenotypic characters are compiled according to *Flora of China*; ^†^ samples of *Quercus robur* are collected from cultivation in Tacheng City, Xinjiang Province, China.

**Table 2:** Estimations of the major clade age with related abiotic events and mean lineage diversification rates for the Chinese oak’s lineage.

Infrageneric Event	Description (Node)	Time Estimation (Ma)	Mean Speciation/Extinction/Turnover Rate (Species/Myr)	Related Climatic/Geological Event ^†^
Clade divergence	New World clade–Old World clade (A)	43.54 (95% HPD: 25.02–64.92)	--	Early uplift of the Qinghai-Tibetan Plateau
	*Cyclobalanopsis*-(*Ilex* + *Cerris*) (B)	38.69	--	Eocene–Oligocene Glaciation
	*Ilex*-*Cerris* (C)	36.04 (95% HPD: 28.37–52.00)	--	Eocene–Oligocene Glaciation
Section diversification	*Cyclobalanopsis* (D)	29.29 (95% HPD: 15.56–46.97)	0.1899/0.0934/0.2833	Oligocene Glaciation
	*Ilex* (E)	30.97	0.1759/0.0865/0.2624	Oligocene Glaciation
	*Cerris* (F)	24.56 (95% HPD: 18.47–30.44)	0.1730/0.0874/0.2604	Late Oligocene Warming/Establishment of Asian Monsoon
	*Quercus* (G)	6.66 (95% HPD: 2.61–15.91)	0.2496/0.1508/0.4004	Miocene cooling/Intensification of Asian Monsoon

HPD: highest posterior density; ^†^ related abiotic events are derived from Zachos et al. (2001) and Favre et al. (2015) [43,44].

## Supplementary Materials

The following are available online at https://www.mdpi.com/2223-7747/9/8/1024/s1, Figure S1: Infrageneric phylogeny of Chinese oak species inferred with (a) maximum likelihood and (b) neighbor-net methods using a concatenate chloroplast dataset. The four identified sections in the Chinese oak’s lineage are color-coded. Gray circles on the branches of the maximum likelihood tree indicate bootstrap values > 60, and a bootstrap confidence of > 60 was shown for major clusters on the neighbor-net network; Figure S2: Infrageneric phylogeny of Chinese oak species inferred with (a) maximum likelihood and (b) neighbor-net methods based on a combined nuclear dataset. The four identified sections in the Chinese oak’s lineage are color-coded. Gray circles on branches of the maximum likelihood tree indicate bootstrap values > 60, and a bootstrap confidence of > 60 was shown for major clusters on the neighbor-net network; Figure S3: Unrooted maximum likelihood trees based on five chloroplast alignments from the Chinese oak species. Individuals are colored according to the four identified sections in the Chinese oak’s lineage; Figure S4: Unrooted maximum likelihood trees based on two nuclear loci from the Chinese oak species. Individuals are colored according to the four identified sections in the Chinese oak’s lineage; Figure S5: Multiple traits-based principal component analysis (PCA) in 46 Chinese oak species; Table S1: Information of seven molecular markers used in this study; Table S2: Genetic estimation of the Chinese oak’s lineage based on chloroplast and nuclear datasets; Table S3: Bonferroni *t*-test of the rates of speciation (lambda) and phenotypic evolution (beta) among four sections of the Chinese oak species; Table S4: Sample fractions of 46 Chinese oak species for a simulation of Bayesian analysis of macroevolutionary mixtures (BAMM); Table S5: Values of phenotypic traits and first two principal components (PCs) in 46 Chinese oak species.
