# Supplementary material for: Framework Phylogeny, Evolution and Complex Diversification of Chinese Oaks
Source: Plants (Basel). 2020 Aug 13;9(8):1024. doi: 10.3390/plants9081024 (PMC7464331; doi:10.3390/plants9081024)
Supplement: Supplementary file 1 [file plants-09-01024-s001.zip › TableS1-S5.docx]

| **Table S1.** Information of seven molecular markers used in this study | | |  |  |  |  |
| --- | --- | --- | --- | --- | --- | --- |
| **Markers** | **Primers** | **Aligned length (bp)** | **N of individuals** | **N of species** | **Evolutionary model** | **MFDM test (P-value)** |
| *ycf*3*-trn*S | F: AAATCGCACCATCTCTGTAATAGG | 509 | 258 | 50 | GTR+F+R3 | 0.2891 |
|  | R: CAAAACCGGGTGAATAGTGAGTC |  |  |  |  |  |
| *mat*K*-trn*K | F: TCTTACGATTTCTGCCCCTTCT | 670 | 256 | 50 | GTR+F+R3 | 0.5984 |
|  | R: TTCTTAGCGGATCGGTTCAAAA |  |  |  |  |  |
| *psb*A*-trn*H | F: GTTATGCATGAACGTAATGCTC | 289 | 260 | 49 | GTR+F+I | 0.2868 |
|  | R: CGCGCATGGTGGATTCACAATCC |  |  |  |  |  |
| *mat*K | F: ACCCAGTCCATCTGGAAATCTTGGTTC | 734 | 260 | 50 | GTR+F+I | 1 |
|  | R: CGTACAGTACTTTTGTGTTTACGAG |  |  |  |  |  |
| *ycf*1 | F: ATTCTGATGGTCCGGAAGGG | 449 | 267 | 50 | GTR+F+R2 | 1 |
|  | R: CCTTATCAGACTGAAACGACTAC |  |  |  |  |  |
| ITS | F: GTTCGGGCGACGGGACAC | 332 | 247 | 49 | GTR+F+R3 | 0.7087 |
|  | R: CCTGCGGGCGGGGACCTC |  |  |  |  |  |
| SAP | F: ATGGAGCATGATGAGACGGG | 355 | 223 | 50 | HKY+F+R2 | 0.5393 |
|  | R: GCCTTAACAACAGGGTTGGC |  |  |  |  |  |

| **Table S2.** Genetic estimation of the Chinese oak lineage based on chloroplast and nuclear datasets | | | | | |  |  |  |  |  |  |
| --- | --- | --- | --- | --- | --- | --- | --- | --- | --- | --- | --- |
|  | **Chloroplast data** |  |  |  |  |  | **Nuclear data** |  |  |  |  |
|  | genus *Quercus* | sect. *Quercus* | sect. *Cerris* | sect. *Ilex* | sect. *Cyclobalanopsis* | | genus *Quercus* | sect. *Quercus* | sect. *Cerris* | sect. *Ilex* | sect. *Cyclobalanopsis* |
| N of individuals | 239 | 75 | 18 | 104 | 42 |  | 205 | 60 | 17 | 88 | 40 |
| N of species | 49 | 12 | 3 | 22 | 12 |  | 49 | 12 | 3 | 22 | 12 |
| Polymorphic sites | 142 | 27 | 4 | 101 | 14 |  | 135 | 61 | 6 | 53 | 46 |
| Parsimony informative sites | 129 | 22 | 1 | 94 | 9 |  | 103 | 48 | 5 | 42 | 29 |
| Gene diversity | 0.9760 | 0.9180 | 0.3860 | 0.9730 | 0.7540 |  | 0.9836 | 0.9870 | 0.8880 | 0.9320 | 0.9470 |
| Nucleotide diversity | 0.0062 | 0.0017 | 0.0002 | 0.0055 | 0.0005 |  | 0.0138 | 0.0088 | 0.0027 | 0.0066 | 0.0083 |
| Theta-W | 0.0089 | 0.0021 | 0.0004 | 0.0073 | 0.0012 |  | 0.0298 | 0.0166 | 0.0021 | 0.0134 | 0.0135 |
| Theta-W: variance of segregating sites | |  |  |  |  |  |  |  |  |  |  |

| **Table S3.** Bonferroni t-test of rates in speciation (lambda) and phenotypic evolution (beta) among four sections of the Chinese oak species | | | | |  |
| --- | --- | --- | --- | --- | --- |
| **Lambda\Beta rates** | ***Quercus*** | ***Cerris*** | ***Ilex*** | ***Cyclobalanopsis*** | |
| *Quercus* | -- | 2.6043NS | 79.1758*** | 51.0560*** | |
| *Cerris* | 50.3117*** | -- | 81.7801*** | 53.6603*** | |
| *Ilex* | 48.3725*** | 1.9392NS | -- | 28.1198*** | |
| *Cyclobalanopsis* | 39.1531*** | 11.1587*** | 9.2195*** | -- | |
| ***P < 0.0001; NS: not significant | |  |  |  | |

| **Table S4.** Sample fractions of 46 Chinese oak species for simulation of Bayesian Analysis of Macroevolutionary Mixtures (BAMM) | | |
| --- | --- | --- |
| **Species** | **Section** | **Fraction** |
| *Q. serrata* var. *brevipetiolata* | *Quercus* | 1.0 |
| *Q. stewardii* | *Quercus* | 1.0 |
| *Q. aliena* var. *acutiserrata* | *Quercus* | 1.0 |
| *Q. griffithii* | *Quercus* | 1.0 |
| *Q. liaotungensis* | *Quercus* | 1.0 |
| *Q. dentata* | *Quercus* | 1.0 |
| *Q. serrata* | *Quercus* | 1.0 |
| *Q. aliena* | *Quercus* | 1.0 |
| *Q. fabri* | *Quercus* | 1.0 |
| *Q. yunnanensis* | *Quercus* | 1.0 |
| *Q. robur* | *Quercus* | 1.0 |
| *Q. mongolica* | *Quercus* | 1.0 |
| *Q. pseudosemecarpifolia* | *Ilex* | 0.9 |
| *Q. engleriana* | *Ilex* | 0.9 |
| *Q. acrodonta* | *Ilex* | 0.9 |
| *Q. oxyphylla* | *Ilex* | 0.9 |
| *Q. baronii* | *Ilex* | 0.9 |
| *Q. phillyraeoides* | *Ilex* | 0.9 |
| *Q. dolicholepis* | *Ilex* | 0.9 |
| *Q. gilliana* | *Ilex* | 0.9 |
| *Q. pannosa* | *Ilex* | 0.9 |
| *Q. semecarpifolia* | *Ilex* | 0.9 |
| *Q. lanata* | *Ilex* | 0.9 |
| *Q. rehderiana* | *Ilex* | 0.9 |
| *Q. spinosa* | *Ilex* | 0.9 |
| *Q. longispica* | *Ilex* | 0.9 |
| *Q. aquifolioides* | *Ilex* | 0.9 |
| *Q. guyavifolia* | *Ilex* | 0.9 |
| *Q. utilis* | *Ilex* | 0.9 |
| *Q. cocciferoides* | *Ilex* | 0.9 |
| *Q. franchetii* | *Ilex* | 0.9 |
| *Q. tarokoensis* | *Ilex* | 0.9 |
| *Q. variabilis* | *Cerris* | 1.0 |
| *Q. chenii* | *Cerris* | 1.0 |
| *Q. acutissima* | *Cerris* | 1.0 |
| *Q. sichourensis* | *Cyclobalanopsis* | 0.2 |
| *Q. gilva* | *Cyclobalanopsis* | 0.2 |
| *Q. arbutifolia* | *Cyclobalanopsis* | 0.2 |
| *Q. glauca* | *Cyclobalanopsis* | 0.2 |
| *Q. oxyodon* | *Cyclobalanopsis* | 0.2 |
| *Q. multinervis* | *Cyclobalanopsis* | 0.2 |
| *Q. myrsinifolia* | *Cyclobalanopsis* | 0.2 |
| *Q. glaucoides* | *Cyclobalanopsis* | 0.2 |
| *Q. sessilifolia* | *Cyclobalanopsis* | 0.2 |
| *Q. championii* | *Cyclobalanopsis* | 0.2 |
| *Q. neglecta* | *Cyclobalanopsis* | 0.2 |

| **Table S5.** Values of phenotypic traits and first two principal components (PCs) in 46 Chinese oak species | | | | |  |  |  |
| --- | --- | --- | --- | --- | --- | --- | --- |
| **Section** | **Species** | **Leaf character^†^** | **Cupule character^‡^** | **Mid-florescence** | **Fruiting period with florescence^§^** | **PC1** | **PC2** |
| *Quercus* | *Q. serrata* var. *brevipetiolata* | 1 | 1 | 3.5 | 1 | -1.3131 | -1.2098 |
|  | *Q. stewardii* | 1 | 2 | 3.5 | 1 | -0.8736 | -1.2965 |
|  | *Q. aliena* var. *acutiserrata* | 1 | 1 | 3.5 | 1 | -1.3131 | -1.2098 |
|  | *Q. griffithii* | 1 | 1 | 4.5 | 1 | -1.4915 | -0.5168 |
|  | *Q. liaotungensis* | 1 | 1 | 4.5 | 1 | -1.4915 | -0.5168 |
|  | *Q. dentata* | 1 | 2 | 4.5 | 1 | -1.0520 | -0.6035 |
|  | *Q. serrata* | 1 | 1 | 3.5 | 1 | -1.3131 | -1.2098 |
|  | *Q. aliena* | 1 | 1 | 4.5 | 1 | -1.4915 | -0.5168 |
|  | *Q. fabri* | 1 | 1 | 4 | 1 | -1.4023 | -0.8633 |
|  | *Q. yunnanensis* | 1 | 2 | 3.5 | 1 | -0.8736 | -1.2965 |
|  | *Q. robur* | 1 | 1 | 3.5 | 1 | -1.3131 | -1.2098 |
|  | *Q. mongolica* | 1 | 1 | 4.5 | 1 | -1.4915 | -0.5168 |
| *Ilex* | *Q. pseudosemecarpifolia* | 2 | 1 | 5.5 | 1 | -0.8246 | 1.2180 |
|  | *Q. engleriana* | 2 | 1 | 4.5 | 1 | -0.6462 | 0.5250 |
|  | *Q. acrodonta* | 2 | 1 | 3.5 | 1 | -0.4678 | -0.1680 |
|  | *Q. oxyphylla* | 2 | 2 | 5.5 | 2 | 0.9421 | 0.9805 |
|  | *Q. baronii* | 3 | 2 | 4 | 2 | 2.1114 | 1.2869 |
|  | *Q. phillyraeoides* | 2 | 1 | 3.5 | 1 | -0.4678 | -0.1680 |
|  | *Q. dolicholepis* | 2 | 2 | 4 | 2 | 1.3043 | -0.0151 |
|  | *Q. gilliana* | 2 | 1 | 5.5 | 1 | -0.8246 | 1.2180 |
|  | *Q. pannosa* | 2 | 1 | 5.5 | 2 | 0.5590 | 0.9802 |
|  | *Q. semecarpifolia* | 2 | 1 | 5.5 | 1 | -0.8246 | 1.2180 |
|  | *Q. lanata* | 2 | 1 | 5.5 | 2 | 0.5590 | 0.9802 |
|  | *Q. rehderiana* | 2 | 1 | 5.5 | 1 | -0.8246 | 1.2180 |
|  | *Q. spinosa* | 2 | 1 | 5.5 | 2 | 0.5590 | 0.9802 |
|  | *Q. longispica* | 2 | 1 | 5.5 | 1 | -0.8246 | 1.2180 |
|  | *Q. aquifolioides* | 2 | 1 | 5.5 | 1 | -0.8246 | 1.2180 |
|  | *Q. guyavifolia* | 2 | 1 | 4.5 | 1 | -0.6462 | 0.5250 |
|  | *Q. utilis* | 2 | 1 | 4.5 | 1 | -0.6462 | 0.5250 |
|  | *Q. cocciferoides* | 2 | 1 | 5.5 | 1 | -0.8246 | 1.2180 |
|  | *Q. franchetii* | 2 | 1 | 2.5 | 1 | -0.2894 | -0.8609 |
|  | *Q. tarokoensis* | 2 | 1 | 4.5 | 2 | 0.7374 | 0.2872 |
| *Cerris* | *Q. variabilis* | 1 | 3 | 3.5 | 2 | 0.9495 | -1.6209 |
|  | *Q. chenii* | 1 | 2 | 3.5 | 2 | 0.5100 | -1.5342 |
|  | *Q. acutissima* | 1 | 3 | 3.5 | 2 | 0.9495 | -1.6209 |
| *Cyclobalanopsis* | *Q. sichourensis* | 2 | 4 | 5.5 | 1 | 0.4939 | 0.9578 |
|  | *Q. gilva* | 2 | 4 | 5 | 1 | 0.5831 | 0.6113 |
|  | *Q. arbutifolia* | 2 | 4 | 0.5 | 2 | 2.7696 | -2.7447 |
|  | *Q. glauca* | 2 | 4 | 4.5 | 1 | 0.6723 | 0.2649 |
|  | *Q. oxyodon* | 2 | 4 | 5.5 | 1 | 0.4939 | 0.9578 |
|  | *Q. multinervis* | 2 | 4 | 5.5 | 2 | 1.8775 | 0.7201 |
|  | *Q. myrsinifolia* | 2 | 4 | 6 | 1 | 0.4047 | 1.3043 |
|  | *Q. glaucoides* | 2 | 4 | 5 | 1 | 0.5831 | 0.6113 |
|  | *Q. sessilifolia* | 2 | 4 | 4.5 | 1 | 0.6723 | 0.2649 |
|  | *Q. championii* | 2 | 4 | 2 | 2 | 2.5019 | -1.7053 |
|  | *Q. neglecta* | 2 | 4 | 2.5 | 2 | 2.4127 | -1.3588 |
| ^†^1-deciduous; 2-evergreen; 3-semievergreen; ^‡^1-ovate bract; 2-lanceolate bract; 3-subulate bract; 4-ring shaped bract; ^§^1-present year; 2-following year | | | | | | | |
